# Polyphenols, Antioxidant Potential and Color of Fortified Wines during Accelerated Ageing: The Madeira Wine Case Study

**DOI:** 10.3390/molecules18032997

**Published:** 2013-03-05

**Authors:** Vanda Pereira, Francisco Albuquerque, Juan Cacho, José C. Marques

**Affiliations:** 1 Centre of Exact Sciences and Engineering, University of Madeira, Campus da Penteada, 9000-390 Funchal, Portugal; E-Mail: marques@uma.pt; 2 Madeira Wine Company, Rua dos Ferreiros, 191, 9000-082 Funchal, Portugal; E-Mail: fma@madeirawinecompany.com; 3 Department of Analytical Chemistry, Faculty of Sciences, University of Zaragoza, 50009 Zaragoza, Spain; E-Mail: jcacho@unizar.es

**Keywords:** polyphenols, antioxidant potential, color, wines, heating, ageing

## Abstract

Polyphenols, antioxidant potential and color of three types of fortified Madeira wines were evaluated during the accelerated ageing, named as *estufagem*. The traditional *estufagem* process was set to 45 °C for 3 months. Overheating conditions, 1 month at 70 °C, were also examined. Total polyphenols (TP), total monomeric anthocyanins (TMA) and total flavonoids (TF) were assessed by spectrophotometric methods, while individual polyphenols and furans were simultaneously determined by HPLC-DAD. Antioxidant potential (AP) was estimated by ABTS, DPPH and FRAP assays, while color was evaluated by Glories and CIELab. Traditional e*stufagem* decreased the TP and AP up to 20% and 26%, respectively, with final values similar to other wines. TMA of the Madeira wines from red grapes decreased during *estufagem*. Six hydroxybenzoic acids, three hydroxycinnamic acids, one stilbene, three flavonols and three flavan-3-ols were found in these wines. The prominent phenolics were hydroxycinnamates and hydroxybenzoates, even after *estufagem*. Most polyphenols decreased, with the exception of caffeic, ferulic, *p*-coumaric, gallic and syringic acids. Finally, both chromatic systems revealed that all wines tended to similar chromatic characteristics after *estufagem*. The study suggests that *estufagem* can be applied without high impact on polyphenols and antioxidant potential of these fortified wines.

## 1. Introduction

Together with aroma and taste, color is an essential feature in the sensory evaluation criteria of wine quality, influencing wine consumer selection. Polyphenols are the main contributors for certain organoleptic characteristics of wines, such as astringency and bitterness, but in particular color. In addition, interest in wine phenolics is still increasing due to their antioxidant and free radical-scavenging proprieties, supported by the health benefits resulting from the moderate wine consumption with respect to cardiovascular diseases, cancer, diabetes, and others [1,2].

Occurrence of phenolics in wine is strongly affected by grape variety and vineyard location (soil, climate and sun exposure), vine cultivation practices, ripening stage at harvesting time and vinification techniques like maceration, fermentation with the grape solids, pressing, sulfite addition, maturation, fining and ageing techniques [3]. Additionally, yeast type and wood-aging can influence the wine polyphenolic content [1]. The most important fraction of wine phenolics is firstly removed from grapes during vinification, mainly from skins but also from seeds, stems and pulp. Wine polyphenols cover flavonoids and non-flavonoids. Flavonoids are characterized by two phenol structures connected by an oxygen-containing carbon-ring structure (a pyran) and comprise flavanols, like quercetin, flavan-3-ols, like catechins, and also anthocyanins and tannins responsible for the red color and mouth-feel of wines, respectively. Flavonoids are the major polyphenols in red wines, constituting more than 85% of the phenolic content and usually found in concentrations ranging from 1,000 to 1,800 mg/L, but less than 20% of these levels in white wines [1,4]. The structurally simpler non-flavonoid constituents include the phenolic, hydroxybenzoic and hydroxycinnamic acids. The levels of phenolic acids in red wines usually range from 100 to 200 mg/L, while in white wines are often lower [5]. Other minor non-flavonoids include volatile phenols (e.g., guaiacol), usually considered responsible for off-flavors, stilbenes (such as *trans*-resveratrol) and miscellaneous compounds like lignans and coumarins [4,6]. It is recognized that non-flavonoids increase the stabilization of red wines, a phenomenon usually related with intra- and intermolecular reactions of anthocyanins [6,7,8]. In general, white wines possess less polyphenols than red wines, with hydroxycinnamates as the major ones. These compounds, namely the esters of tartaric acid (caftaric and coutaric acids) together with some flavanols, like (+)-catechin and (−)-epicatechin, are considered the major oxidation substrates and browning precursors in white wines, to form yellow-brown products due to the polymerization of *o*-quinones [9,10]. Flavanols react with other flavanols through direct or acetaldehyde- and glyoxylic acid-mediated condensations [11]. In the case of red wines, free anthocyanins are progressively transformed into more stable oligomeric and polymeric pigments, since the beginning of the vinification and ageing. Indeed, anthocyanins condense with flavanols (catechins), directly or mediated by acetaldehyde, and with yeast metabolites (essentially pyruvic acid) to form pyranoanthocyanins [12]. The reactivity of polyphenols increases the complexity due to the variety of the resulting compounds and has an important effect on the sensorial properties of wines, especially on color due to wine browning, but also in taste and colloidal stability during storage and ageing [13].

Besides sensory attributes, antioxidant potential can presumably be affected by the oxidation of polyphenolic compounds in wines that develop non-enzymatic browning [10]. It would be expected that oxidation of phenolics could lead to a lower antioxidant capacity, but this is not necessarily true as novel polyphenolic compounds might be produced.

In recent years, several studies were published concerning the effect of the ageing on color and phenolic content especially in red wines, including the wood effect [13,14,15,16,17,18] and ageing in bottle [19]. Especially in the case of white wines, the attention has been centered in browning due to polyphenolic oxidation, considered as an undesirable occurrence in table wines [10,20,21,22,23,24,25,26]. In contrast, browning of Madeira wines is seen as a pleasant phenomenon, associated to quality and typical characteristics. These fortified wines (17%–22% of ethanol) produced on Madeira Island, Portugal, are obtained from both white and red approved varieties of *Vitis vinifera* L. Madeira wine is submitted to an oxidative ageing process, which favors their specific sensory features: from pale yellow color, typical of dry wines, to dark brown, characteristic of sweet wines; and very complex aromas. Most of these wines are submitted to *estufagem* (heating for three months, usually up to 50 °C, in a closed vat), an accelerated ageing process, before undergoing the normal maturation process in oak casks at wine cellar lofts (*canteiro*), usually for a period of three years. The acquired characteristic taste and complex bouquet together with the browning color can be associated to oxidation and eventual sugar degradation through Maillard type reactions, acidic degradation or even caramelization, especially in sweet wines [27]. Considering the limited information available, the main purpose of the present study was to determine the influence of the heating step in the polyphenolic composition, antioxidant potential and in the color development. The study involved the estimation of color, total phenolics, antioxidant potential and individual polyphenolic composition of sweet and dry wines produced from *Tinta Negra Mole* (*TNM*) grapes (red variety), and a traditional sweet wine produced from *Malvasia* grapes (white variety). The wines were fortified and heated at 45 °C for a 3 month period. Additionally, in order to evaluate the temperature effect, the wines were also submitted to overheating conditions, at 70 °C during 1 month, temperature high enough to reinforce the oxidative process without the wine boil.

## 2. Results and Discussion

The experiments were focused on the evolution of the polyphenolic composition, antioxidant potential, and color of Madeira wines submitted to the traditional accelerated ageing, with the purpose of establishing the influence of temperature on this class of compounds. 

### 2.1. Polyphenolic Composition

The effect of the accelerated ageing on the polyphenolic composition was evaluated in total terms by spectrophotometric measurements and individual polyphenols by HPLC-DAD. Table 1 summarizes the attained results in terms of total polyphenols (TP), total monomeric anthocyanins (TMA) and total flavonoids (TF). The results show that young wines produced from *TNM* grapes (red wines) present similar levels of TP when compared with the white variety *Malvasia*. This may be related to the fact that the fermentation of Madeira wines is usually performed as in white table wines, in the absence (or limited contact) of grape solids. Ageing can also affect the content of phenols, as they can suffer hydrolysis, oxidations and complexations, while temperature is known to increase degradation [28]. Actually, Folin-Ciocalteu’s assay (frequently used to assess the TP) reveal that the process of *estufagem* promoted some changes on the phenolic composition, but did not greatly affect the total content of polyphenols of the Madeira wines submitted to this procedure. 

Although a maximum decrease of 25% was found in the 70 °C experiment with *Malvasia* wine, the variations obtained for the other two wines in the same experiment were not significant (*p* < 0.050). Under standard conditions (45 °C for three months) it was observed that the maximum loss was smaller and even non-significant in *TNM* sweet (Table 1). These results showed that temperature did not produce a consistent effect and that *estufagem* can be applied without a high impact on the polyphenolic composition After the heating step at 45 °C, the TP was found to vary from 617.10 to 492.16 mg(GAE)/L in *Malvasia*, and from 469.98 to 434.42 and 609.98 to 493.09 mg(GAE)/L in sweet and dry *TNM*, respectively (Table 1). TP final values range from 434.42 to 573.57 mg(GAE)/L, which were comparable with those presented in the literature for white wines [29,30,31], or slightly higher [32,33,34].

**Table 1:** Total polyphenols (TP), total flavonoids (TF) and total monomeric anthocyanins (TMA) in Madeira wines at the initial stage and at the end of each month of heating at 45 °C (3 months) and 70 °C (1 month).

*Samples*		*TP mg(GAE)/L*	*± SD*	*TMA mg(Cyd-3-glu)/L*	*± SD*	*TF QE(mg/L)*	*± SD*
***TNM* sweet**	**0 m**	469.98***ab***	13.63	15.02***a***	0.01	28.96***a***	0.39
	**1 m, 45 °C**	332.17***c***	9.58	4.43***b***	0.03	23.47***b***	1.55
	**2 m, 45 °C**	474.15***b***	15.64	3.16***c***	0.03	38.06***c***	0.33
	**3 m, 45 °C**	434.42***a***	9.03	0.93***d***	0.02	30.70***d***	0.54
	**1 m, 70 °C**	444.01***ab***	17.21	1.62***e***	0.05	51.44***e***	0.60
***TNM* dry**	**0 m**	609.98***a***	25.83	22.05***a***	0.05	49.42***a***	0.56
	**1 m, 45 °C**	576.68***ab***	23.60	2.99***b***	0.10	45.97***ab***	0.31
	**2 m, 45 °C**	561.02***b***	4.17	5.05***c***	0.02	45.80***b***	0.25
	**3 m, 45 °C**	493.09***c***	3.04	2.50***d***	0.02	45.39***b***	0.16
	**1 m, 70 °C**	573.57***ab***	6.37	0.30***e***	0.04	82.87***c***	2.92
***Malvasia***	**0 m**	617.10***a***	7.22	-		28.48***a***	0.11
	**1 m, 45 °C**	565.11***ab***	47.84	-		33.60***a***	0.32
	**2 m, 45 °C**	517.12***bc***	18.02	-		46.48***b***	1.60
	**3 m, 45 °C**	492.16***bd***	40.92	-		47.76***b***	0.48
	**1 m, 70 °C**	466.02***cd***	18.06	-		133.74***c***	4.18

Different letters in the same column means significant differences at *p* < 0.050.

Although only responsible for up to 4% of the phenolics of *TNM* wines, anthocyanins may also contribute to the decrease of the TP. The loss of anthocyanins was proved by the progressive decrease of the total monomeric anthocyanins (TMA) obtained in *TNM* wines (Table 1). The initial TF values were in general very low, ranging from 28.48 to 49.42 mg(QE)/L, as result of the small skin contact in winemaking. These values were close to those obtained by Mitić *et al.* [33] (between 45 and 81 mg/L, as catechin equivalents) when they analyzed 10 Serbian white wines using the same test. The flavonoids do not represent more than 8% of the total polyphenolic content of the wines at the initial circumstances and, surprisingly, the aluminium chloride assays revealed that TF values increased with the heating period, especially when overheating temperature was applied. This was not expected since it is frequently referred that flavonoids participate in several reactions, namely anthocyanin and flavanols degradation [11], which could induce the decrease on the TF values. Eventually, other substances with capacity of reducing aluminium (III) are formed during this period and respond positively to this test. Regarding the HPLC-DAD analysis, sixteen polyphenols were identified in the current sample set of Madeira wines (Table 2, Table 3 and Table 4), including non-flavonoids (six hydroxybenzoic acids, three hydroxycinnamic acids and one stilbene) and flavonoids (three flavonols and three flavan-3-ols). Additionally, two furans were also identified—5-hydroxymethylfurfural (HMF) and furfural.

**Table 2:** Individual polyphenols and furans (mg/L) in *TNM* sweet wine during heating at 45 °C (3 months) and 70 °C (1 month).

		*TNM sweet*									
		*0 m*	*± SD*	*1 m, 45* *°C*	*± SD*	*2 m, 45* *°C*	*± SD*	*3 m, 45* *°C*	*± SD*	*1 m, 70* *°C*	*± SD*
***Non-flavonoids***											
*Hydroxybenzoics*											
	Gallic acid	3.70***a***	0.01	4.91***b***	0.07	5.29***c***	0.03	6.23***d***	0.12	9.16***e***	0.09
	Protocatechuic acid	2.57***a***	0.11	2.35***b***	0.06	1.97***c***	0.02	1.55***d***	0.08	1.64***d***	0.05
	*p*-Hydroxybenzoic acid	0.94***a***	0.05	0.57***b***	0.02	0.77***c***	0.03	0.51***b***	0.02	1.00***a***	0.06
	Vanillic acid	2.52***a***	0.09	2.57***a***	0.06	2.23***b***	0.06	2.24***b***	0.11	3.08***c***	0.08
	Syringic acid	4.89***a***	0.06	5.29***b***	0.08	5.03***a***	0.18	5.03***a***	0.06	5.93***c***	0.04
	Ellagic acid	n.d.		n.d.		n.d.		n.d.		n.d.	
	**Total**	**14.62**		**15.69**		**15.29**		**15.56**		**20.81**	
*Hydroxycinnamates*											
	*trans*-Caftaric acid	14.53***a***	0.01	13.37***b***	0.01	11.31***c***	0.02	10.82***d***	0.05	3.24***e***	0.04
	*cis*-Coutaric acid	4.45***a***	0.04	3.71***b***	0.02	2.91***c***	0.01	2.73***d***	0.11	0.62***e***	0.02
	*trans*-Coutaric acid	7.87***a***	0.02	7.66***b***	0.01	7.07***c***	0.03	6.67***d***	0.15	2.99***e***	0.06
	*trans*-Fertaric acid	0.91***a***	0.02	0.82***b***	0.02	0.71***c***	0.02	0.78***b***	0.01	1.80***d***	0.05
	Unk 5 *	2.20***a***	0.04	2.57***b***	0.10	2.34***c***	0.03	2.40***c***	0.03	2.70***d***	0.02
	Caffeic acid	1.72***a***	0.02	1.82***a***	0.07	2.06***b***	0.02	2.53***c***	0.10	3.34***d***	0.15
	Unk 6 *	1.96***a***	0.05	2.07***b***	0.02	1.91***a***	0.01	2.05***b***	0.02	2.09***b***	0.01
	*p*-Coumaric acid	0.58***a***	0.01	0.85***b***	0.02	1.02***c***	0.01	1.27***d***	0.01	4.31***e***	0.09
	Ferulic acid	n.q.		0.45***a***	0.02	0.47***a***	0.00	0.68***b***	0.03	0.45***a***	0.02
	**Total**	**34.22**		**33.32**		**29.81**		**29.93**		**21.52**	
*Stilbene*											
	*trans*-Resveratrol	n.d.		n.d.		n.d.		n.d.		n.d.	
***Flavonoids***											
*Flavonols*											
	Unk 7 **	8.03***a***	0.07	7.13***b***	0.06	5.71***c***	0.09	5.30***d***	0.04	0.56***e***	0.03
	Myricetin	n.d.		n.d.		n.d.		n.d.		n.d.	
	Quercetin	n.q.		n.q.		n.q.		0.53a	0.01	0.67b	0.01
	Kaempferol	n.q.		n.q.		n.q.		n.q.		n.q.	
	**Total**	**8.03**		**7.13**		**5.71**		**5.83**		**1.23**	
*Flavan-3-ols*											
	*(−)*-Epigallocatechin	n.d.		n.d.		n.d.		n.d.		n.d.	
	*(+)*-Catechin	3.94***a***	0.02	2.49***b***	0.05	1.48***c***	0.02	0.93***d***	0.03	0.47***e***	0.00
	*(−)*-Epicatechin	0.92***a***	0.01	0.65***b***	0.03	0.47***c***	0.03	0.40***d***	0.01	0.36***d***	0.01
	**Total**	**4.86**		**3.13**		**1.95**		**1.32**		**0.84**	
***Furans***											
	HMF	1.56***a***	0.02	17.90***b***	0.00	49.32***c***	0.03	97.50***d***	0.12	1728.07***e***	2.80
	Furfural	n.d.		n.q.		2.02a	0.05	3.08b	0.05	20.26c	0.18
	**Total**	**1.56**		**17.90**		**51.34**		**100.58**		**1748.32**	

* Quantification relative to caffeic acid; ** Quantification relative to rutin; n.d. - not detected, bellow LOD; n.q. - not quantified, bellow LOQ; HMF - 5-hydroxymethylfurfural; Different letters in the same row (in ***bold***) means that there are significant differences at *p* < 0.050.

**Table 3:** Individual polyphenols and furans (mg/L) in *TNM* dry wine during heating at 45 °C (3 months) and 70 °C (1 month).

		*TNM dry*									
		*0 m*	*± SD*	*1 m, 45 °C*	*± SD*	*2 m, 45 °C*	*± SD*	*3 m, 45 °C*	*± SD*	*1 m, 70 °C*	*± SD*
***Non-flavonoids***											
*Hydroxybenzoics*											
	Gallic acid	9.47***a***	0.37	9.81***a***	0.01	9.91***a***	0.14	9.50***a***	0.05	10.44***b***	0.08
	Protocatechuic acid	6.84***a***	0.39	3.53***b***	0.15	2.47***c***	0.14	2.19***c***	0.09	4.74***d***	0.06
	*p*-Hydroxybenzoic acid	1.15***a***	0.02	1.31***b***	0.05	1.22***ab***	0.06	1.28***ab***	0.04	1.67***c***	0.06
	Vanillic acid	4.92***a***	0.17	3.81***b***	0.11	3.42***c***	0.03	3.23***c***	0.06	3.27***c***	0.08
	Syringic acid	3.39***a***	0.16	3.72***b***	0.05	4.27***c***	0.09	4.11***c***	0.08	4.90***d***	0.04
	Ellagic acid	n.q.		n.q.		n.q.		n.q.		n.q.	
	**Total**	**25.77**		**22.18**		**21.29**		**20.31**		**25.02**	
*Hydroxycinnamates*											
	*trans*-Caftaric acid	37.34***a***	0.14	32.78***b***	0.02	27.63***c***	0.01	27.64***c***	0.04	5.71***d***	0.02
	*cis*-Coutaric acid	5.14***a***	0.02	4.37***b***	0.05	3.69***c***	0.04	3.67***c***	0.00	0.64***d***	0.01
	*trans*-Coutaric acid	20.55***a***	0.15	19.60***b***	0.04	17.37***c***	0.08	17.32***c***	0.01	5.03***d***	0.12
	*trans*-Fertaric acid	n.d.		n.d.		n.d.		n.d.		n.d.	
	Unk 5 *	2.20***a***	0.06	2.66***b***	0.01	2.68***b***	0.03	2.66***b***	0.03	2.93***c***	0.01
	Caffeic acid	2.75***a***	0.09	4.29***b***	0.03	5.18***c***	0.04	5.21***c***	0.04	6.00***d***	0.02
	Unk 6 *	1.63***a***	0.03	2.19***b***	0.03	2.19***b***	0.08	2.19***b***	0.06	2.62***c***	0.03
	*p*-Coumaric acid	1.61***a***	0.03	2.31***b***	0.05	2.78***c***	0.04	2.77***c***	0.00	8.32***d***	0.02
	Ferulic acid	0.45***a***	0.01	0.66***b***	0.02	0.66***b***	0.01	0.65***b***	0.02	0.51***c***	0.02
	**Total**	**71.69**		**68.85**		**62.19**		**62.12**		**31.76**	
*Stilbene*											
	*trans*-Resveratrol	0.63a		0.51b	0.01	n.q.		n.q.		n.q.	
***Flavonoids***											
*Flavonols*											
	Unk 7 **	6.13***a***	0.03	4.93***b***	0.07	4.04***c***	0.06	3.98***c***	0.10	0.46***d***	0.01
	Myricetin	0.71***a***	0.03	0.75***a***	0.01	0.72***a***	0.01	n.q.		1.15***b***	0.03
	Quercetin	0.82***a***	0.01	0.89***b***	0.01	0.81***a***	0.01	0.81***a***	0.01	1.53***c***	0.01
	Kaempferol	n.q.		n.q.		n.q.		n.q.		n.q.	
	**Total**	**7.66*a***		**6.57**		**5.56**		**4.79**		**3.15**	
*Flavan-3-ols*											
	(−)-Epigallocatechin	3.54***a***	0.12	3.18***b***	0.06	1.47***c***	0.04	1.41***c***	0.05	n.d.	
	(+)-Catechin	16.19***a***	0.02	13.06***b***	0.06	6.16***c***	0.06	6.20***c***	0.04	4.27***d***	0.11
	(−)-Epicatechin	4.78***a***	0.18	3.21***b***	0.06	1.46***c***	0.07	1.30***c***	0.06	1.07***d***	0.03
	Total	24.52		19.45		9.08		8.91		5.33	
***Furans***											
	HMF	n.d.		1.61***a***	0.01	2.70***b***	0.03	2.68***b***	0.03	41.28***c***	0.05
	Furfural	n.d.		n.q.		1.66***a***	0.01	1.65***b***	0.00	12.20***c***	0.02
	**Total**	**0.00**		**1.61**		**4.36**		**4.33**		**53.47**	

* Quantification relative to caffeic acid; ** Quantification relative to rutin; n.d. - not detected, bellow LOD; n.q. - not quantified, bellow LOQ; HMF - 5-hydroxymethylfurfural; Different letters in the same row (in ***bold***) means that there are significant differences at *p* < 0.050.

**Table 4:** Individual polyphenols and furans (mg/L) in *Malvasia* wine during heating at 45 °C (3 months) and 70 °C (1 month).

		*Malvasia*									
		*0 m*	*± SD*	*1 m, 45 °C*	*± SD*	*2 m, 45 °C*	*± SD*	*3 m, 45 °C*	*± SD*	*1 m, 70 °C*	*± SD*
***Non-flavonoids***											
*Hydroxybenzoics*											
	Gallic acid	11.62***a***	0.42	13.73***bc***	0.07	13.85***b***	0.02	13.27***cd***	0.08	12.93***d***	0.12
	Protocatechuic acid	3.40***a***	0.01	2.67***b***	0.15	2.66***b***	0.03	2.95***c***	0.05	3.48***a***	0.15
	*p*-Hydroxybenzoic acid	0.92***a***	0.03	0.88***a***	0.04	0.68***b***	0.03	0.61***c***	0.01	0.90***a***	0.01
	Vanillic acid	1.71***a***	0.03	0.93***b***	0.04	0.66***c***	0.01	0.57***d***	0.02	0.55***d***	0.01
	Syringic acid	n.d.		n.d.		n.d.		0.93***a***	0.05	0.75***b***	0.03
	Ellagic acid	n.d.		n.d.		n.d.		n.d.		n.d.	
	**Total**	**17.66**		**18.21**		**17.85**		**18.33**		**18.61**	
*Hydroxycinnamates*											
	*trans*-Caftaric acid	37.33***a***	0.07	30.47***b***	0.02	23.31***c***	0.02	16.87***d***	0.02	7.25***e***	0.04
	*cis*-Coutaric acid	7.40***a***	0.03	6.32***b***	0.02	5.28***c***	0.31	3.91***d***	0.13	0.93***e***	0.00
	*trans*-Coutaric acid	15.51***a***	0.08	13.27***b***	0.06	10.76***c***	0.01	8.20***d***	0.04	4.88***e***	0.00
	*trans*-Fertaric acid	3.08***a***	0.05	2.71***b***	0.16	2.14***c***	0.03	1.60***d***	0.02	0.86***e***	0.02
	Unk 5 *	0.62***a***	0.01	0.48***b***	0.01	n.q.		n.q.		n.d.	
	Caffeic acid	1.71***a***	0.03	3.10***b***	0.02	3.60***c***	0.02	3.55***d***	0.05	6.72***e***	0.02
	Unk 6 *	n.d.		n.d.		n.d.		n.d.		n.d.	
	*p*-Coumaric acid	0.76***a***	0.01	1.35***b***	0.00	1.63***c***	0.02	1.65***c***	0.05	7.72***d***	0.09
	Ferulic acid	0.37***a***	0.02	0.66***b***	0.01	0.72***c***	0.04	0.62***b***	0.01	1.00***d***	0.02
	**Total**	**66.77**		**58.36**		**47.42**		**36.39**		**29.36**	
*Stilbene*											
	*trans*-Resveratrol	n.q.		n.q.		n.q.		n.d.		n.d.	
***Flavonoids***											
*Flavonols*											
	Unk 7 **	7.25***a***	0.07	6.80***b***	0.11	5.31***c***	0.18	3.79***d***	0.06	0.48***e***	0.03
	Myricetin	n.d.		n.d.		n.d.		n.d.		n.d.	
	Quercetin	0.64***a***	0.01	0.96***b***	0.01	0.85***c***	0.00	0.65***a***	0.01	0.75***d***	0.01
	Kaempferol	n.q.		n.q.		n.q.		n.q.		n.q.	
	**Total**	**7.89**		**7.76**		**6.17**		**4.44**		**1.24**	
*Flavan-3-ols*											
	*(−)*-Epigallocatechin	0.55***a***	0.01	n.q.		n.q.		n.q.		0.60***b***	0.02
	*(+)*-Catechin	6.98***a***	0.05	4.23***b***	0.06	1.25***c***	0.03	0.49***d***	0.02	n.q.	
	*(−)*-Epicatechin	1.47***a***	0.04	0.90***b***	0.02	0.49***c***	0.03	0.39***d***	0.01	0.37***d***	0.02
	Total	9.00		5.12		1.74		0.88		0.97	
***Furans***											
	HMF	1.48***a***	0.00	12.17***b***	0.01	30.42***c***	0.01	52.10***d***	0.15	1651.50***e***	2.78
	Furfural	n.d.		n.q.		1.80***a***	0.02	2.33***b***	0.05	19.48***c***	0.01
	**Total**	**1.48**		**12.17**		**32.22**		**54.43**		**1670.98**	

* Quantification relative to caffeic acid; ** Quantification relative to rutin; n.d. - not detected, bellow LOD; n.q. - not quantified, bellow LOQ; HMF - 5-hydroxymethylfurfural; Different letters in the same row (in ***bold***) means that there are significant differences at *p* < 0.050.

Moreover, the most important unknown peaks were tentatively identified, by the elution order and UV spectrum when compared with those found in literature. The first six unknown peaks exhibit cinnamic-type UV spectra, and it is believed that they correspond to hydroxycinnamates, currently found in wines. In fact, evidences indicate that some of them are hydroxycinnamoyltartaric acids. These compounds were identified by Buiarelli *et al.* [35] in wine by using HPLC-tandem mass spectrometry. Using a standard C_18_-column, they established the following elution order: caftaric, coutaric, fertaric, caffeic, *p*-coumaric and ferulic. Darias-Martín *et al.* [36] also reported that the *cis* forms elute before *trans* ones. Consequently, comparing the UV spectra with those obtained by Guerrero *et al.* [37], Mozetič *et al.* [38] and Gutiérrez *et al.* [18], Unk 1 (maximum at 326 nm with a shoulder at 300 nm) was identified as *trans*-caftaric acid, Unk 2 and Unk 3 as *cis*- and *trans*-coutaric acids, with maximums at 310 and 313 nm, respectively, and Unk 4 as fertaric acid (maximum at 327 nm with a shoulder at 287 nm), probably the *trans* form, the most common in wines. Unk 5 and 6 should correspond to the hydroxycinnamates family, but their identification was not established. Unk 7 has a UV spectrum similar to rutin (standard available) and may have structural similarities.

Before heating, hydroxycinnamates represent on average 59% of the non-anthocyanin polyphenols, followed by hydroxybenzoic acids (about 20%). Caftaric acid was the most abundant compound found in all wines, varying from 14.53 (Table 2) to 37.34 mg/L (Table 3) (caffeic acid equivalents). Similar amounts were found by Fernández-Pachón and colleagues [39] in sherry wines (6.29 to 42.90 mg/L). During the heating step, a noticeable decrease of caftaric, coutaric and fertaric acids was registered, especially when heating conditions were more severe. Conversely, caffeic, *p*-coumaric and ferulic contents improved during the same period and with the temperature increase, suggesting the hydrolysis of the correspondent hydroxycinnamoyltartaric acids (Table 2, Table 3 and Table 4).

Flavan-3-ols, initially ranging from 0.55 mg/L for (−)-epigallocatechin (Table 3) to 16.19 mg/L for (+)-catechin (Table 3), also progressively declined during the heating process. Similar findings have also been pointed out by others researchers during wine standard ageing [10,21,38]. The decrease of hydroxycinnamic acid esters and flavanols—yellow pigments [4]—due to oxidation is referenced to contribute to the development of the brownish shades in white wines, although flavanols have been considered more effective in browning, especially (−)-epicatechin [13].

Some hydroxybenzoic acids increased during the heating period (gallic and syringic acid) and others declined (protocatechuic, *p*-hydroxybenzoic and vanillic acids). Gallic acid was the major hydroxybenzoate and its growth during ageing is usually explained by the hydrolysis of gallic tannins [6,40]. The values found (3.70 to 13.85 mg/L) are slightly above of those found by Darias-Martín *et al.* [36] in white wines from the Canary Islands (0.97 to 1.64 mg/L), but similar to those reported by Fernández-Pachón *et al.* [39] in sherry wines (4.42 to 10.70 mg/L). Syringic acid increase during ageing is usually related with the anthocyanins cleavage or the breakdown of lignin during wine wood-ageing [41], which could explain the result obtained for *TNM* wines (Table 2 and Table 3). The degradation of the others hydroxybenzoates may be related with the formation of ethyl esters of vanillic and *p*-hydroxybenzoic acids, and methyl esters of vanillic and protocatechuic acids, already found in wines [6]. Only trace amounts of ellagic acid were detected. 

Flavonols, common in the skins of both red and white grapes in glycoside form, with the aglycone form prevailing in wines, were found but represent a small fraction (less than 13%). The results were consistent with the absence or limited contact with grape solids during fermentation, and the highest values were found in *TNM* dry wines. Unk 7 is important and decreases with heating. Quercetin was found in small amounts (ranging from 0.53 to 1.53 mg/L) and did not present a regular trend with the heating process, like myricetin, only found in the *TNM* dry wine (about 0.71 mg/L). Traces of kaempferol were found, but below the quantification limit and *trans*-resveratrol (not detected in sweet wines and up to 0.63 mg/L in *TNM* dry wine) was clearly affected by temperature (Table 2, Table 3 and Table 4). This value is within the range of 0.1–0.8 mg/L found in white wines [6]. On the other hand, HMF and furfural levels, formed essentially from sugar degradation, are clearly improved with temperature, as pointed out in a previous work [42], especially HMF in the sweet wines (Table 2, Table 4). The low levels obtained for the dry wine (less than 3 mg/L, Table 3), except in overheating conditions, suggest the need of accurate control of the temperature used in the *estufagem,* and eventually different heating temperatures should be considered according to the wine sweetness.

Generally speaking, the HPLC-DAD results showed that the content of simple (non-polymeric polyphenols) non-anthocyanin polyphenols, present in the studied Madeira wines, diminish significantly during the *estufagem* process. The decline at standard conditions (45 °C, 3 months) represents in average 27% while the overheating experiment (70 °C, 1 month) promoted a decrease of about 43% (Table 5), revealing that temperature potentiate the transformation of this kind of phenolics. The obtain data also demonstrated that flavonoids decrease had a preponderant effect on this result. Table 5 also indicates that non-flavonoids are the most abundant polyphenols in these wines, even after *estufagem*.

**Table 5:** Total of polyphenols (mg/L) during the heating at 45 °C (3 months) and 70 °C (1 month) of the studied Madeira wines, regarding HPLC-DAD analysis.

*Samples*		*Total polyphenols (mg/L)*	*Dec. %*	*Non-flavonoids (mg/L)*	*Dec. %*	*Flavonoids (mg/L)*	*Dec. %*
***TNM* sweet**	**0 m**	61.73	-	48.84	-	12.89	-
	**1 m, 45 °C**	59.29	4.0	49.02	-0.4	10.27	20.4
	**2 m, 45 °C**	52.76	14.5	45.10	7.6	7.66	40.6
	**3 m, 45 °C**	52.63	14.7	45.49	6.9	7.15	44.6
	**1 m, 70 °C**	44.39	28.1	42.33	13.3	2.06	84.0
***TNM* dry**	**0 m**	130.27	-	98.09	-	32.18	-
	**1 m, 45 °C**	117.55	9.8	91.53	6.7	26.02	19.1
	**2 m, 45 °C**	98.12	24.7	83.48	14.9	14.64	54.5
	**3 m, 45 °C**	96.13	26.2	82.43	16.0	13.70	57.4
	**1 m, 70 °C**	65.26	49.9	56.78	42.1	8.48	73.6
***Malvasia***	**0 m**	101.32	-	84.43	-	16.89	-
	**1 m, 45 °C**	89.44	11.7	76.56	9.3	12.88	23.7
	**2 m, 45 °C**	73.18	27.8	65.27	22.7	7.91	53.2
	**3 m, 45 °C**	60.04	40.7	54.72	35.2	5.32	68.5
	**1 m, 70 °C**	50.18	50.5	47.97	43.2	2.21	86.9

Dec.% - Decrease percentage.

### 2.2. Antioxidant Potential

The antioxidant potential (AP) was determined by three different tests: ABTS, DPPH and FRAP, and the results are listed in Table 6. The DPPH assay presented the highest values (on average 382.22 mg(Trolox)/L) and the FRAP the lowest (about 55.39 mg(QE)/L). FRAP assay measures the reducing capacity of antioxidants and depends totally on the electron transference mechanisms while DPPH and ABTS assays determines the radical scavenging activity by electron and hydrogen transfer [43]. DPPH (R^2^ = 0.725) and ABTS (R^2^ = 0.7411) assays likely reflect better the AP of the studied wines rather than FRAP assay (R^2^ = 0.1158), since the correlation with the TP is higher. When HPLC-DAD results (total polyphenols) are correlated with the different antioxidant assays, it is observed that DPPH (R^2^ = 0.813) traduce better the AP rather than ABTS assay (R^2^ = 0.645), while the antioxidant activity measured by the FRAP assay does not correspond to the identified phenolics, since it is not found any type of correlation (R^2^ = 0.000). It's evident that other antioxidants than phenols react in the FRAP system, hypothesizing the presence of non-phenolic antioxidants.

**Table 6:** Antioxidant potential of Madeira wines during the heating at 45 °C (3 months) and 70 °C (1 month) expressed in terms of ABTS, DPPH and FRAP assays.

*Samples*		*ABTS assay*		*DPPH assay*				*FRAP assay*	
		mg(GAE)/L	± SD	mg(Trolox)/L	± SD	IC_50_ (µL)	± SD	mg(QE)/L	± SD
***TNM* sweet**	**0 m**	150.76***a***	1.16	313.99***a***	12.34	49.99***a***	2.56	40.65***a***	2.18
	**1 m, 45 °C**	103.07***b***	2.52	276.66***b***	13.27	65.51***b***	0.86	19.88***b***	0.69
	**2 m, 45 °C**	174.07***c***	3.16	308.73***a***	16.45	50.93***a***	1.51	51.50***c***	2.37
	**3 m, 45 °C**	153.93***a***	3.50	305.52***a***	2.36	53.85**a**	2.39	53.49***c***	0.92
	**1 m, 70 °C**	149.82***a***	1.23	234.84***c***	5.37	68.90***b***	3.49	42.98***a***	0.29
***TNM* dry**	**0 m**	268.61***a***	0.13	502.93***a***	19.46	25.68***a***	0.49	36.83***a***	3.22
	**1 m, 45 °C**	249.74***b***	9.13	496.66***a***	13.50	25.84***a***	0.78	28.67***b***	2.42
	**2 m, 45 °C**	235.36***c***	8.50	504.98***a***	9.22	27.06***b***	0.28	33.67***ab***	1.90
	**3 m, 45 °C**	198.45***d***	7.25	409.66***b***	9.42	36.79***c***	0.31	102.46***c***	4.31
	**1 m, 70 °C**	218.21***e***	6.32	403.53***b***	5.13	34.30***d***	0.68	120.27***d***	1.86
***Malvasia***	**0 m**	183.93***a***	4.95	445.92***a***	7.00	32.56***a***	0.73	70.21***a***	3.39
	**1 m, 45 °C**	214.76***b***	9.34	426.01***a***	3.55	34.67***a***	0.55	75.30***b***	1.25
	**2 m, 45 °C**	219.29***b***	8.08	389.33***b***	22.98	39.07***b***	0.65	60.76***c***	2.55
	**3 m, 45 °C**	177.06***a***	2.85	362.99***bc***	6.36	43.10***c***	0.45	55.33***c***	3.89
	**1 m, 70 °C**	144.65***c***	0.39	351.60***c***	6.39	60.54***d***	2.79	38.81***d***	1.85

The results showed that changes occurred in the AP during the heating step do not follow a typical trend. A decrease of up to 26% (ABTS assay) was observed after *estufagem* under standard conditions (45 °C, 3 months), while similar results were observed after heating a 70 °C in only 1 month. Taking into account that *estufagem* is an accelerated oxidative process, this decrease do not cause a negative impact enough to change this procedure. The above results clearly show that the changes observed in polyphenols by HPLC-DAD might indicate the formation of other antioxidants with ageing.

At the end of the heating procedure the AP values were in the range 234.84–409.66 mg/L in terms of TEAC (or 0.94–1.64 mM) slightly above of the results obtained by Fernández-Pachón *et al.* [39] in sherry wines (0.49–0.98 mM), and comparable to those found by de Quirós *et al.* [44] in Spanish white wines (0.77–2.01 mM).

### 2.3. Color Study

**Figure 1:**
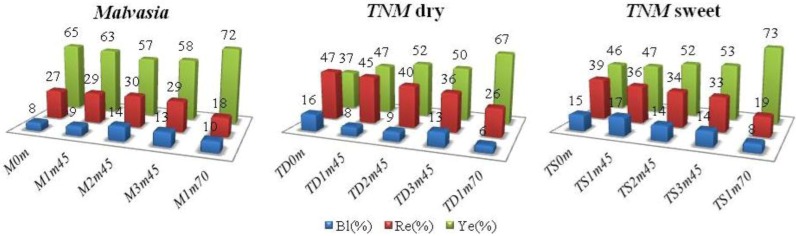
Glories parameters, *%Ye*, *%Re* and *%Bl*, of Madeira wines during the heating at 45 °C (3 months) and 70 °C (1 month). **Sample identification:** *M*, *TD* and *TS* stand for *Malvasia*, *TNM* dry and *TNM* sweet wines, respectively, while the following two digits correspond to the heating period and the last two represent the heating temperature.

As color is one of the principal attributes of a wine and it is considered decisive for the choice of consumers, color studies can be a helpful tool in the recognition of the typical characteristics of a wine or on the influence of the vinification procedures. As Madeira wines can present pronounced color changes during *estufagem*, Glories and CIELab systems were applied. Glories parameters, *%Ye*, *%Re* and *%Bl*, are presented in Figure 1 while color intensity (*CI*), tonality (*To*) and the absorbance reading at 420 nm are reported in Table 7.

**Table 7:** Glories chromatic parameters: intensity (*CI*), tonality (*To*) and absorbance readings at 420 nm of Madeira wines submitted to heating at 45 °C (3 months) and 70 °C (1 month).

*Wines*		*A_420_*	*± SD*	*CI*	*± SD*	*To*	*± SD*
***TNM* sweet**	**0 m**	1.435***a***	0.033	3.14***a***	0.09	1.17***a***	0.00
	**1 m, 45 °C**	1.840***b***	0.117	3.93***b***	0.30	1.31***b***	0.01
	**2 m, 45 °C**	1.174***c***	0.120	2.27***c***	0.27	1.52***c***	0.03
	**3 m, 45 °C**	1.167***c***	0.052	2.19***c***	0.13	1.63***d***	0.03
	**1 m, 70 °C**	2.149***d***	0.019	2.94***a***	0.05	3.87***e***	0.08
***TNM* dry**	**0 m**	1.580***a***	0.019	4.22***a***	0.06	0.80***a***	0.00
	**1 m, 45 °C**	1.064***b***	0.004	2.27***b***	0.01	1.05***b***	0.00
	**2 m, 45 °C**	1.311***c***	0.007	2.53***c***	0.02	1.31***c***	0.00
	**3 m, 45 °C**	1.743***d***	0.043	3.46***d***	0.10	1.39***d***	0.00
	**1 m, 70 °C**	0.948***e***	0.003	1.41***e***	0.00	2.55***e***	0.00
***Malvasia***	**0 m**	0.692***a***	0.001	1.07***a***	0.00	2.36***a***	0.00
	**1 m, 45 °C**	1.070***b***	0.004	1.71***b***	0.01	2.19***b***	0.00
	**2 m, 45 °C**	1.253***c***	0.030	2.21***c***	0.06	1.92***c***	0.01
	**3 m, 45 °C**	0.859***d***	0.011	1.47***d***	0.03	2.05***d***	0.02
	**1 m, 70 °C**	2.135***e***	0.016	2.97***e***	0.05	4.03***e***	0.11

Different letters in the same column means significant differences at *p* < 0.050.

The results show that before heating, red color (about 47%) predominates in *TNM* dry wine (*TD0m*), while the yellow tones characterize the studied sweet wines, especially *Malvasia* (*M0m*) with 65% (Figure 1) and limited contribution of blue hue (up to 16%). The heating process clearly affects color, expressed in the increase of *%Ye* (reaches about 35% and 15% in the dry and sweet wine submitted to heating at 45 °C for 3 months, *TD3m45* and *TS3m45*, respectively) and the decrease of *%Re* (intensified at overheating temperatures, up to 81% in *TD1m70* and 59% in *TS1m70*). The decrease of the reddish and the increase of the yellowish shades are in agreement with the observations reported by other authors [15,19] and can be associated with the degradation of anthocyanins to form new polymeric complexes. Indeed, the anthocyanin degradation was confirmed by the TMA analysis (Table 1). In *Malvasia* wine, the yellow pigments were always preponderant (at least 57%), as expected for a white variety. *TNM* wines presented high *CI* values before heating (Table 7) which decreased with *estufagem*, following the decreasing of red hues, while *To* slightly increased. Almost the opposite was observed for *Malvasia*. 

Usually used as a browning index in white wines, the absorbance at 420 nm did not reveal a consistent trend during the heating period (45 °C), but increased significantly in sweet wines under overheating conditions (70 °C). These results are in agreement with those of Mayén *et al.* [20] who found that A_420_ did not increase during the browning of white wines from Pedro Ximenez and Baladi grapes, at accelerated ageing (50 °C) in corked bottles, but however, increased when the bottles were opened and exposed to air. On the other hand, Kallithraka *et al.* [10] reported that A_420_ of white wines significantly increased only after accelerated ageing at 55 °C over a period of 10 days. In addition, Fernandez-Zurbano *et al.* [21] established three categories for the browning of white dry wines, considering intense when absorbance (AU) was higher than 0.5, moderate between 0.2 and 0.5 AU and light when less than 0.2 AU was registered. Considering these categories, Madeira wines present an intense browning at the end of the thermal processing, especially when sweet wines were heated at 70 °C, for 1 month (Table 7). *TNM* wines at the initial stage already present very high A_420_ values, possibly due to the presence of anthocyanins and others phenolics. At overheating conditions, reactions between polyphenolic compounds and sugars degradation, namely caramelization, might be favored. 

**Figure 2:**
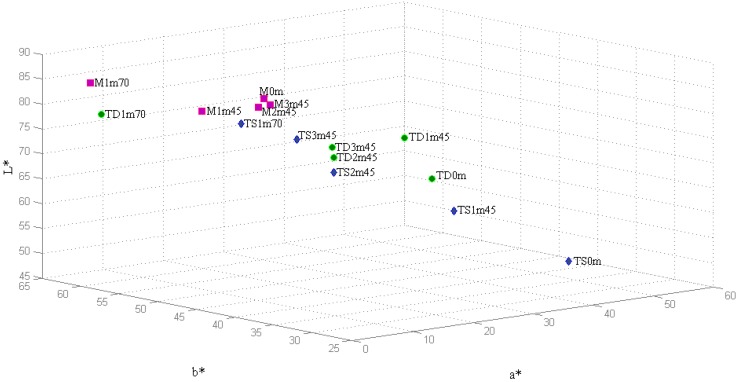
3D representation of the CIELab chromatic coordinates a*, b* and L* of the Madeira wines submitted to heating at 45 °C (3 months) and 70 °C (1 month). **Sample identification:** *M*, *TD* and *TS* stand for *Malvasia*, *TNM* dry and *TNM* sweet wines, respectively, while the following two digits correspond to the heating period and the last two represent the heating temperature.

The CIELab chromatic coordinates a*, b* and L* were also obtained and are represented in a 3D plot (Figure 2). Color differences were more noticeable than with the Glories procedure, however confirmed the conclusions. 

Major changes were observed again in *TNM* dry wine, reflecting the decrease on the a* positive coordinate (red hue) associated with an increase of the b* positive values (yellow hue), more pronounced at overheating conditions. 

The variations with the heat presented by *TNM* sweet wine and *Malvasia* were significantly smaller, except for overheating conditions. Figure 2 clearly shows that all wines tend to the same chromatic characteristics when the heating procedure is applied: red wines become clearer (L* increases) due to anthocyanin polymerization, while yellow tones (b* increases) predominate rather than red (a* decreases). No defined trend was observed for chromaticity (C*, ranging from 32.53 to 63.46 units), hue (H*, ranging from 0.62 and 1.52 units) and saturation (S*, ranging from 0.40 to 1.32 units) during the heating period (Table 8).

**Table 8:** CIELab chromatic parameters: chromaticity (C*), hue (H*) and saturation (S*) of the Madeira wines submitted to heating at 45 °C (3 months) and 70 °C (1 month).

*Samples*		C *	*± SD*	H *	*± SD*	S *	*± SD*	ΔE *
***TNM* sweet**	**0 m**	47.05	0.00	0.91	0.00	0.69	0.00	-
	**1 m, 45 °C**	32.58	0.00	1.12	0.00	0.40	0.00	-
	**2 m, 45 °C**	49.85	0.00	1.11	0.00	0.70	0.00	-
	**3 m, 45 °C**	41.80	0.00	1.21	0.00	0.54	0.00	16.45
	**1 m, 70 °C**	63.33	0.00	1.46	0.00	0.82	0.00	34.90
***TNM* dry**	**0 m**	63.46	0.00	0.62	0.00	1.32	0.00	-
	**1 m, 45 °C**	58.18	0.00	0.82	0.00	0.99	0.00	-
	**2 m, 45 °C**	62.56	0.00	1.01	0.00	0.98	0.01	-
	**3 m, 45 °C**	55.32	0.00	1.14	0.00	0.75	0.00	40.21
	**1 m, 70 °C**	51.36	0.00	1.31	0.00	0.65	0.00	50.55
***Malvasia***	**0 m**	41.11	0.00	1.43	0.00	0.47	0.00	-
	**1 m, 45 °C**	54.88	0.00	1.35	0.00	0.68	0.00	-
	**2 m, 45 °C**	43.16	0.00	1.41	0.00	0.51	0.00	-
	**3 m, 45 °C**	40.64	0.00	1.42	0.00	0.47	0.00	1.34
	**1 m, 70 °C**	61.46	0.00	1.52	0.00	0.72	0.00	20.96

All results were significantly different at *p* < 0.050.

Differences detectable by the human eye were estimated by the measurement of colorimetric differences (ΔE*) for every pair of wines after the heating period, as follows: ΔE* = [(ΔL*)^2^ + (Δa*)^2^ + (Δb*)^2^]^1/2^, assuming that ΔE* higher than 3 units means that the color of the samples is different enough to be easily distinguished by human observers [45]. Results (Table 8) revealed that the color of the wines after being heated was clearly distinguishable from the initial stage (16.45 < ΔE* < 50.55), with the exception of *Malvasia*, under standard conditions.

## 3. Experimental

### 3.1. Wine Samples

Two *Vitis vinifera* L. grapes varieties recommended for the production of Madeira wine, *Tinta Negra Mole* (*TNM*, red variety) and *Malvasia* (white variety), were used in the study. The grapes were collected during the 2007 harvest and the corresponding wines were elaborated by a local Madeira wine-producing cellar. Sulfite addition was set to about 60 mg/L into grape juice. The alcoholic fermentation was conducted under controlled temperature without maceration or adding commercial yeasts. Two types of wines were produced from *TNM* grapes in adequate stainless steel tanks, stopping the fermentation by the addition of natural grape spirit when the density of grape must reached 1,025 g/cm^3^ (115 g/L of reducing sugars) or 986 g/cm^3^ (about 4 g/L of reducing sugars) for sweet and dry *TNM* wine, respectively. The sweet *Malvasia* wine was prepared in a similar way and the fermentation was stopped when the density reached 1,019 g/cm^3^ (about 96 g/L of reducing sugars). After fortification, each wine was forced-aged in a special pilot scale system equipped with 200 L stainless steel tanks fitted with heating coils, allowing the circulation of pre-heated tap water, and maintained at 45 °C during 3 months. For comparison purposes, each wine was also submitted to overheating conditions, 70 °C during 1 month. All wines were monthly sampled (three sample replicates) and kept at −20 °C before analysis. 

### 3.2. Chemicals

Folin-Ciocalteu reagent and gallic acid (≥98.0%) were supplied by Fluka Biochemika AG (Buchs, Switzerland) while sodium carbonate (99.8%), potassium chloride and sodium acetate (≥99%) were from Panreac Química S.A. (Barcelona, Spain). Aluminium chloride-6-hydrate and quercetin (≥99%) were from Riedel de Haën (Seelze, Germany) and methanol HPLC gradient grade was supplied by Fisher Scientific (Loughborough, UK).

For the preparation of the PBS buffer solution the following chemicals were used: sodium chloride, potassium chloride, sodium hydroxide, di-sodium hydrogen phosphate 12-hydrate and potassium di-hydrogen phosphate, supplied by Panreac Química S.A. (≥98%). 2,2'-Azino-bis-(3-ethylbenzothiazoline-6-sulfonic acid) diammonium salt (ABTS, 98.0%) and potassium persulfate (≥98.0%) were supplied by Fluka Biochemika AG.

For the DPPH assay, 2,2-diphenyl-1-picrylhydrazyl (DPPH^•^, 90.0%) and Trolox (6-hydroxy-2,5,7,8-tetramethylchloromane-2-carboxylic acid, ≥ 98%) were obtained from Fluka Biochemika AG. For the FRAP assay, 2,2-dipyridyl (99%), trichloroacetic acid (TCA, ≥99.0%), sodium tartrate (≥99.0%) and citric acid monohydrate (≥99.5%) were purchased from Fluka Biochemika AG while iron chloride (≥99%) was supplied by Riedel de Haën. 

The following polyphenolic standards (purity higher than 95%) were used: gallic acid, vanillic acid, caffeic acid, *p*-coumaric acid, ferulic acid, ellagic acid, *p*-hydroxybenzoic acid, (+)**-catechin, (−)-epicatechin, (−)-epigallocatechin, myricetin, rutin and kaempferol from Fluka Biochemika AG, protocatechuic acid, vanillin, syringic acid and *trans*-resveratrol from Sigma-Aldrich (St. Louis, MO, USA), syringaldehyde, HMF and furfural were from Acros Organics (Geel, Belgium) and quercetin from Riedel-de-Haën.

### 3.3. Polyphenolic Composition

Wines were assayed for total polyphenols (TP, gallic acid equivalents, mg/L), total monomeric anthocyanins (TMA, cyanidin-3-glucoside equivalents, mg/L) and total flavonoids (TF, quercetin equivalents, mg/L).

TP was determined following the Folin-Ciocalteu’s method adopted from the International Organization of Vine and Wine [46], using gallic acid as standard. Briefly, to 100 µL of wine/calibration standards were added the following reagents: 5 mL of distilled water, 0.5 mL of Folin–Ciocalteu reagent and 2 mL of 20% (w/v) sodium carbonate aqueous solution, bringing the volume to 10 mL with distilled water and then the sample was mixed. The absorbance at 750 nm was measured after 30 min. The concentration of the phenolic compounds was calculated according to the following gallic acid calibration curve: A_750_ = 0.0008 GAE(mg/L) + 0.0058 (R^2^ = 0.999), set for the range of 100 to 800 mg/L.

The TMA was determined using the pH-differential method proposed by AOAC (Official Method 2005.02 [47]). This determination was only performed for the red wines. Wine samples were tentatively diluted in 0.025 M potassium chloride buffer pH 1.0 until the absorbance at 510 nm was lower than unity. The samples were then diluted (1:5) in 0.4 M sodium acetate buffer pH 4.5. Absorbance readings at 510 and 700 nm in each buffer were performed, using distilled water as blank. The concentration of TMA was then calculated with the following formula and expressed as cyanidin-3-glycoside (cyd-3-glu) equivalents (mg/L): TMA = (A × MW × DF × 10^3^)/(*ε* × l), with A = (A_520_ − A_700_)_pH 1.0_ − (A_520_ − A_700_)_pH 4.5_, where MW is the cyd-3-glu molecular weight (449.2 g/mol), DF is the dilution factor, l is the path length cell (1 cm), *ε* is cyd-3-glu molar absorptivity coefficient (26,900 L/mol cm) and 10^3^ a conversion factor (g to mg).

The TF was determined according to the aluminium chloride colorimetric method proposed by Meda *et al.* [48], with small adjustments: 5 mL of 2% (w/v) aluminium chloride (AlCl_3_) solution in methanol was mixed with the same volume of wine/standard. Absorbance readings at 415 nm were carried out after 10 min of incubation at room temperature, using methanol as blank. The concentrations of TF were calculated according to the obtained equation of the standard quercetin calibration graph (10–50 mg/L): A_415_ = 0.0240 QE (mg/L) − 0.0100 (R^2^ = 0.999).

All determinations were performed in a Perkin-Elmer Lambda 2 spectrophotometer (Waltham, MA, USA) using 1 cm quartz cells. The average of the relative standard deviation (%RSD) among replicates was 14%.

Individual polyphenols were determined by direct injection of the samples in a high performance liquid chromatography (HPLC) system, following the methodology described by Pereira *et al.* [49], with slight modifications. Briefly, the chromatographic system (Waters Alliance, Milford, MA, USA) was equipped with an auto-injector (Waters 2695, separations module), a photodiode array detector (Waters 2996) and the Empower Pro software, for data handling. The polyphenolic compounds were separated in an Atlantis T_3_ column (250 × 4.6 mm, i.d.; 5 μm; Milford, MA, USA) using the gradient described in Table 9, based on the following solvents: A (10 mM of phosphate buffered at pH 2.70), B (acetonitrile) and C (methanol), and setting the column temperature to 30 °C. 20 μL of each sample/standard were injected after filtration using 0.45 µm Acrodisc^®^ GHP filters (Pall Gelman Sciences, Ann Arbor, MI, USA). 

**Table 9:** Gradient program of polyphenols. Mobile phases: A – 10 mM of phosphate buffered at pH 2.70; B – acetonitrile; C – methanol.

*Time (min)*	*Flow (mL/min)*	*%A*	*%B*	*%C*	*Curve*
---	1.00	100.0	0.0	0.0	---
30.00	1.00	79.0	10.0	11.0	6
42.00	1.00	73.0	10.0	17.0	6
55.00	1.00	40.0	60.0	0.0	6
58.00	1.00	40.0	60.0	0.0	6
65.00	1.00	100.0	0.0	0.0	1

All standards and wine samples were injected in triplicate. Detection was performed at specific wavelengths after scanning from 200 to 780 nm. The identification of the analytes was carried out by comparing retention times and spectra with those of original standards, when available. All others were tentatively identified based on spectra obtained from the literature and assayed by assuming similar molar absorptivities to compounds with structural similarities. Quantitative determinations were attempted using external standard calibration method. Wavelengths used for quantification were 210 nm for flavan-3-ols and benzoic acids, 280 nm for furans, 315 nm for hydroxycinnamic acids and *trans*-resveratrol and 360 nm for flavonoids and ellagic acid. 

### 3.4. Antioxidant Potential

The antioxidant potential was estimated by different methods: ABTS, DPPH and FRAP. All photometric measurements were carried out in triplicate, with RSD values below 9%. 

The antioxidant potential was firstly measured according to the ABTS assay based on Re *et al.* [50] with some modifications, using gallic acid as antioxidant standard. Firstly, a phosphate buffered saline (PBS) solution was prepared as follows: 8.18 g NaCl, 0.27 g KH_2_PO_4_, 3.94 g Na_2_HPO_4_·12H_2_O and 0.15 g KCl in 1 L of distilled water. Then, a 2 mM ABTS^•+^ stock solution was prepared by reacting the ABTS salt with 200 µL of 70 mM potassium persulfate in 50 ml of PBS and allowing the mixture to stand in the dark at room temperature for 16 h before use. The ABTS^•+^ stock solution was then diluted with PBS to obtain an absorbance value of 0.800 ± 0.030 at 734 nm. Finally, 12 µL of sample were mixed with 3 mL of the ABTS^•+^ working solution, and absorbance measurements were performed at room temperature during 20 min, at every 60 seconds, using PBS as blank. The antioxidant power was calculated as percentage of inhibition (%I = [(A_734(0 min)_ − A_734(20 min)_)/A_734(0 min)_] × 100, with A_734(0 min)_ as the absorbance of the ABTS^•+^ at 734 nm at t = 0 min, A_734(20 min)_ the absorbance of the remaining radical at the end of the reaction (t = 20 min)), converted into gallic acid equivalents (GAE) by means of the following calibration curve (50-240 mg/L), submitted to the same procedure described above: %I = 0.328 GAE(mg/L) + 11.638 (R^2^ = 0.996). The antioxidant potential was also evaluated by the ability of wines to scavenge DPPH free radicals, adapting the methodology proposed by Paixão *et al.* [31], and expressing the results as inhibitory concentration IC_50_ and as Trolox^®^ equivalent antioxidant capacity (TEAC). Aliquots of 6, 12, 18, 24, 36 and 60 µL of wine sample were individually mixed with 2.5 mL of DPPH^•^ (60 µM in methanol, daily prepared) and analyzed immediately. The absorbance of the remaining DPPH^•^ was determined after 20 min, in 30 sec periods, at 515 nm, using methanol as blank. The IC_50_ determination was achieved by plotting the DPPH^•^ linear regression in the range 6 to 60 µM: A_515_ = 0.0121[DPPH^•^] − 0.0223 (R^2^ = 0.992), calculating the percentage of remaining DDPH^•^ as follow: %[DPPH^•^]_REM_ = [DPPH^•^]_(20 min)_/[DPPH^•^]_(0 min)_ × 100 and fitting the best curve to the graph sample concentration (µL) against %[DPPH^•^]_REM_. Finally, the IC_50_ parameter was calculated for each sample as the substrate concentration to produce 50% reduction of the DPPH radicals. To express the TEAC, Trolox® concentration was plotted *vs.* %I: %I = 0.0752 TE(mg/L) + 1.9269 (R^2^ = 0.999), with %I = [(A_515(0 min)_ − A_515(20 min)_)/A_515(0 min)_] × 100, and A_515(0 min)_ as the absorbance measured at the beginning of the reaction and A_515(20 min)_ the absorbance after 20 min of reaction of 18 µL of the Trolox® standards (25–1,250 mg/L) following the above procedure.

The ferric reducing/antioxidant power (FRAP) was also performed. The current determination was based on the protocol established by Makris *et al.* [51], using ferric chloride (3 mM FeCl_3_ in 5 mM citric acid) as oxidant and measuring the colored ferrous product formed with 2,2'-dipyridyl at 525 nm. Briefly, 250 µL of working FRAP solution, daily prepared, was mixed with 250 µL of sample properly diluted, and then mixed with 4.5 mL of 0.5% 2,2'-dipyridyl in 1.2% TCA, after a 20-min incubation at 50 °C in a water bath. After 5 min, the FRAP values were obtained from the absorbance recordings at 525 nm and expressed as quercetin equivalents determined from the linear regression set from 3 to 60 mg/L A_525_ = 0.0099 QE(mg/L) + 0.3893 (R^2^ = 0.999), and introducing the dilution factor. 

### 3.5. Color

The color of the wines submitted to *estufagem* was determined by means of the chromatic Glories and CIELab parameters. The wine samples were filtered by cellulose membranes prior to the spectrophotometric analysis. The Glories parameters, yellow percentage (*%Ye*), red percentage (*%Re*), blue percentage (*%Bl*), color intensity (CI) and tonality (T), were determined at 420, 520 and 620 nm. These chromatic indexes are currently used by oenologists, though the CIELab space defines better the wine color and differentiation [52,53]. Thus, the CIELab parameters (L*, a*, b*) were also determined measuring the transmittance from 380 to 770 nm at 5 nm intervals, following the recommendations of OIV [46] and considering the illuminant D_65_ (daylight source) and 10° standard observer (human perception). The a* and b* coordinates correspond to reddish/greenish and yellowish/bluish colors, respectively, and the color lightness, L*, is evaluated in a black and white scale (ranging from 0 to 100). The psychophysical parameters C*, H* and S* were also estimated [54], where the chromaticity (C*) was calculated as C* = [(a*)^2^ + (b*)^2^]^1/2^, the hue (H*) was considered as H* = arctan (b*/a*), while the saturation (S*) was determined as S* = C*/L*.

### 3.6. Data Processing

All analyses were performed in triplicate and the results were expressed as the mean value ± standard deviation. Significant differences were assessed through the analysis of variance (one-way ANOVA with Holm-Sidak method), using the statistical software SigmaPlot 12.0.

## 4. Conclusions

The developed work showed that the accelerated ageing process did not greatly affect the total content of polyphenols of the current fortified wines traditionally submitted to this procedure, only causing a maximum decrease of 25%. At least 434.42 mg(GAE)/L of the total polyphenols were present after *estufagem*, which is comparable with most white wines. After the thermal processing, the antioxidant potential (0.94–1.64 mM) was also similar to most white table wines. In terms of individual polyphenols (non-anthocyanic), six hydroxybenzoic acids, three hydroxycinnamic acids, one stilbene, three flavonols and three flavan-3-ols were found in these wines, with hydroxycinnamates and hydroxybenzoates being the most abundant phenolics. Most individual polyphenols decreased during *estufagem*, except caffeic, ferulic, *p*-coumaric, gallic and syringic acids.

Finally, the color of the wines tended to the same chromatic characteristics when the heating procedure was applied, even red wines become clearer, with yellow tones becoming predominant, as monomeric anthocyanins gradually declined. Browning index values (absorbance at 420 nm) did not reveal a consistent trend during the heating period, but increased significantly at overheating conditions, especially for sweet wine, indicating a probable relation between Madeira wine browning and sugar degradation.

Since the vinification of these fortified wines is up to date mainly based on tradition, the current study give an important contribute to understand the impact of the *estufagem* process on its characteristics, giving valuable information for wine producers improve the Madeira winemaking and further the wine quality. The most important result of the current study was that *estufagem* can be applied without a high impact on the polyphenolic composition and also on the antioxidant potential of these wines.
